# Comparative predictive performance of three machine learning algorithms for acute radiation enteritis risk among patients with cervical cancer undergoing radiotherapy: A prospective cohort study

**DOI:** 10.1016/j.apjon.2026.100853

**Published:** 2026-01-17

**Authors:** Zhao Wang, Huiying Liu, Xiaocen Chen, Fang Zhang, Yixuan Liu, Jiayun Sun, Lili Liu, Xiaotong Yang

**Affiliations:** a Department of Radiotherapy, Tianjin Medical University Cancer Institute & Hospital, National Clinical Research Center for Cancer, Tianjin's Clinical Research Center for Cancer, Key Laboratory of Cancer Prevention and Therapy, Tianjin, China; b Graduate School, Tianjin University of Traditional Chinese Medicine, Tianjin, China

**Keywords:** Machine learning, Acute radiation enteritis, Cervical cancer, Risk prediction, Random forest

## Abstract

**Objective:**

To develop a machine learning-based risk prediction model for acute radiation enteritis (ARE) in patients with cervical cancer, providing a new method for early and accurate prediction of ARE during radiotherapy.

**Methods:**

This prospective study enrolled patients with cervical cancer undergoing radiotherapy from March 2024 to March 2025. The patients were randomly divided into training and test sets at a 7:3 ratio. Prediction models were constructed using Logistic Regression (LR), Decision Tree (DT), and Random Forest (RF) algorithms. Model performance was evaluated based on the area under the receiver operating characteristic curve (AUC), accuracy, precision, sensitivity, specificity, and F1-score.

**Results:**

The incidence of ARE was 52.85% (204/386). Among the three models, the Random Forest model demonstrated the best performance, with an AUC of 0.961, sensitivity of 0.934, and F1-score of 0.905. These performance metrics were consistently higher than those of the LR (AUC, 0.860; sensitivity, 0.739; F1-score, 0.736) and DT (AUC, 0.910; sensitivity, 0.887; F1-score, 0.873) models. The RF model showed good clinical utility in effectively identifying high-risk patients for early intervention. Feature importance ranking derived from the RF model identified the parametrial dose, radiotherapy time, clinical stage, rectal V40, age, Platelet-to-Lymphocyte Ratio (PLR), concurrent chemotherapy, and hypertension as the most influential predictors, in descending order of importance.

**Conclusions:**

The RF-based risk prediction model exhibited excellent performance in assessing the risk of ARE among patients with cervical cancer undergoing radiotherapy, thereby enabling individualized risk assessment and facilitating early preventive strategies.

## Introduction

Recent data (from 2022) from the National Cancer Center predicts 150,700 new cases of cervical cancer in China, with 55,700 deaths, making cervical cancer the most common malignant tumor of the female reproductive tract.^1^ Both the incidence and age-standardized mortality rates are increasing, posing significant challenges to women’ s fertility and overall health.^2^

Radiotherapy is a crucial treatment option for cervical cancer.^3^ Although it effectively kills cancer cells, it inevitably affects the surrounding normal tissues within the radiation range.^4^ Radiation enteritis (RE) is a common complication of this treatment, with clinical manifestations such as abdominal pain, diarrhea, tenesmus, and hematochezia. In severe cases, it can even lead to intestinal obstruction, intestinal fistulae, and sepsis.^5^ Intestinal tissue is sensitive to radiation, causing an imbalance in intestinal epithelial secretion and absorption and leading to acute radiation enteritis (ARE).^6^ ARE is the most common complication, occurring mostly during radiotherapy or within 3 months after completion,^5^ with a high clinical incidence rate.^7^ Studies have shown that ARE not only significantly reduces patients' quality of life but may also force treatment interruption in severe cases, affecting the overall therapeutic effect.^8^^,^^9^ Therefore, early prediction of ARE risk in patients with cervical cancer undergoing radiotherapy can greatly help reduce its incidence and improve patients’ quality of life.

In recent years, numerous studies have identified various risk factors associated with the occurrence of ARE, including general patient characteristics such as age,^10^, ^11^, ^12^, ^13^, ^14^, ^15^, ^16^ body mass index (BMI)^17^; tumor-related factors such as clinical stage,^10^^,^^13^^,^^16^ tumor diameter,^13^^,^^16^ and invasion depth^13^; radiotherapy-related factors such as radiotherapy time,^18^ parametrial tissue radiation dose,^14^^,^^16^ rectal V40,^14^^,^^15^^,^^17^^,^^19^ number of radiotherapy sessions,^13^ and radiotherapy position^17^; comorbidities such as hypertension,^7^^,^^10^^,^^11^^,^^14^ hyperglycemia,^7^^,^^10^^,^^11^ anemia,^10^ malnutrition^19^; as well as laboratory and inflammatory indicators such as Lactate Dehydrogenase-to-Albumin Ratio (LAR),^11^ Platelet-to-Lymphocyte Ratio (PLR),^19^ Lymphocyte-to-Monocyte Ratio (LMR).^19^ However, no single factor has been proven to be sufficient to predict the occurrence of ARE. Given the complexity of radiation-induced injuries, a single risk factor remains inadequate to accurately predict its development. Therefore, there is an urgent need to develop a multifactorial prediction model to guide personalized intervention strategies for patients with cervical cancer undergoing radiotherapy.

Current risk-prediction models exhibit certain limitations. Some studies focused solely on elderly patients^11^ or were constrained by limited sample sizes.^20^^,^^21^ Furthermore, several investigations have employed only traditional statistical methods, such as Logistic Regression (LR), for model construction,^10^^,^^19^, ^20^, ^21^ typically presenting only a single optimal model without a systematic comparison of different modeling algorithms. With the growing application of Machine Learning (ML) in the field of medical prediction, more sophisticated algorithms such as Random Forest (RF) and Decision Tree (DT) have demonstrated the potential to handle nonlinear relationships and complex interactions.^22^, ^23^, ^24^, ^25^ Several studies have indicated that machine learning models can offer superior discriminative performance compared with traditional methods such as LR.^26^, ^27^, ^28^ The adoption of more advanced ML techniques holds promise for further enhancing the model predictive performance and improving the identification of high-risk populations.

Therefore, this study aimed to move beyond the traditional paradigm of single-model construction by systematically introducing and comparing the performance of the RF, DT, and LR algorithms in predicting ARE risk for the first time, thereby identifying an optimal prediction model and enhancing the identification of high-risk patients. The findings will provide empirical evidence to support subsequent researchers in selecting appropriate modeling approaches, offer a basis for clinical staff to develop decision-support tools, and ultimately establish a scientific foundation for the prevention of ARE in patients with cervical cancer undergoing radiotherapy, as well as provide professional support for formulating targeted prevention and control strategies.

## Methods

### Study and sample

**Fig. 1:**
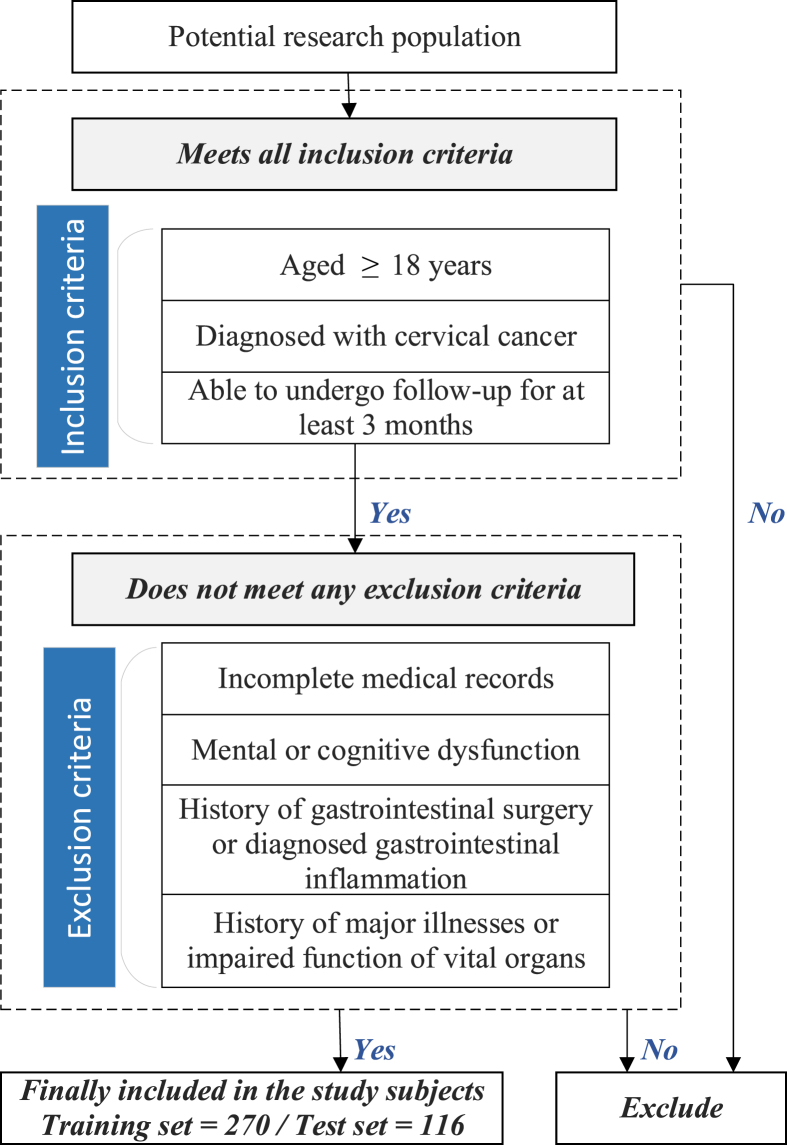
Screening flowchart for research subjects.

This prospective cohort study enrolled patients diagnosed with cervical cancer by convenience sampling in accordance with the 2022 National Comprehensive Cancer Network (NCCN) Clinical Practice Guidelines^29^ using convenience sampling. Participants were recruited from the radiotherapy ward of a tertiary Grade A oncology hospital in Tianjin between March 2024 and March 2025. The specific inclusion and exclusion criteria are summarized in Fig. 1.

**Fig. 2:**
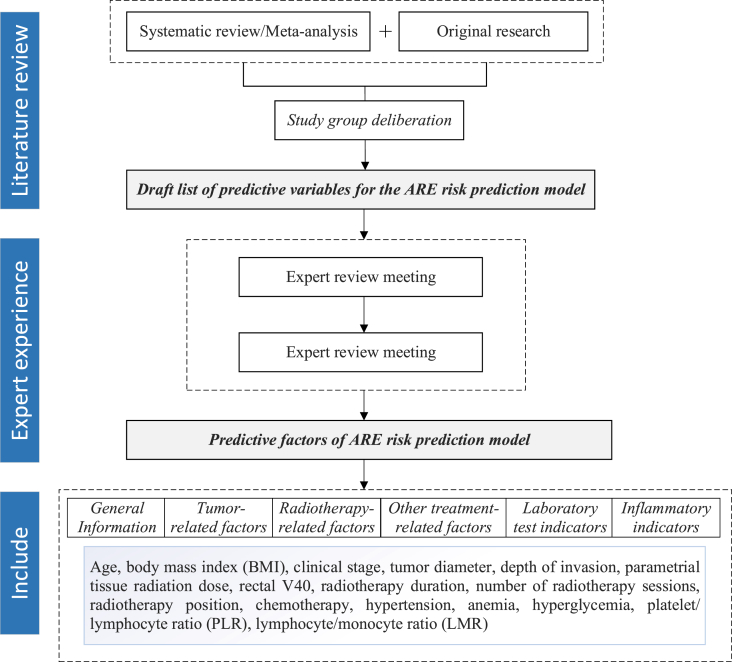
Predictive factor determination process for the ARE risk prediction model.

This study employed the Event Per Variable (EPV) method to estimate the sample size. According to the recommendations of the Prediction Model Risk of Bias Assessment Tool (PROBAST),^30^ the sample size for a prediction model development study should meet a minimum of 10 endpoint events per predictor variable (i.e., EPV ≥ 10). Sixteen candidate predictors were identified (Fig. 2). A literature review indicated that the incidence of ARE in patients with cervical cancer undergoing radiotherapy is 65.2%.^7^ Accounting for an estimated 10% loss to follow-up, the required sample size was calculated using the following equation:

$$N=\left(10\times \frac{K}{I}\right)\times \left(1+10\%\right)$$

where K represents the number of predictor variables, and I represents the incidence rate. By substituting these values (K=16 and I=0.652), the minimum required total sample size was calculated to be 269. The training set of this study ultimately included 270 patients, fulfilling the sample size requirements for model development. Following a conventional 7:3 ratio, the total cohort was randomly split into training and test sets, with the test set comprising 116 patients.

### Criteria for the diagnosis of ARE

This study focused on the occurrence of ARE, defined as intestinal inflammation occurring during or within three months of pelvic radiotherapy.^31^ The diagnosis was based on the RTOG acute radiation morbidity scoring criteria^32^ (Table 1). To ensure diagnosis objectivity and consistency, we implemented standardized prospective data collection and adjudication processes. During radiotherapy, all patients had their gastrointestinal symptoms recorded daily by research nurses using a structured form. Furthermore, the attending physicians conducted comprehensive daily clinical visits with each patient. These visits included detailed history-taking and systematic abdominal physical examination to identify objective clinical signs associated with ARE (such as abdominal tenderness or changes in bowel sounds). Within three months after the completion of radiotherapy, outpatient follow-up visits were conducted on a weekly basis. Patients were instructed to seek medical evaluation at any time if symptoms occurred.

**Table 1:** Radiation Therapy Oncology Group grading criteria for acute radiation enteritis.

Grade	Description
0	No symptoms
1	Mild diarrhea and cramps, with 5 bowel movements per day, excessive rectal mucus, or intermittent bleeding
2	Moderate diarrhea, with colicky bowel movements more than 5 times per day, excessive rectal mucus, or intermittent bleeding
3	Obstruction or bleeding requiring surgical intervention
4	Necrosis, perforation, and sinus formation

The final diagnosis and grading of the ARE were independently determined by two senior radiation oncologists. They comprehensively reviewed all available information: (1) the daily symptom checklist recorded by research nurses, (2) clinical visit notes from attending physicians (including findings from physical examinations), and (3) the patients’ medical records. Based on this information, the two physicians independently diagnosed and graded according to the RTOG criteria. In case of disagreement, another senior expert was consulted for arbitration to reach a final consensus.

### Medical record data collection form

The initial list of potential risk factors for this study was drafted based on existing primary research and systematic reviews^7^^,^^10^, ^11^, ^12^, ^13^, ^14^, ^15^, ^16^, ^17^, ^18^, ^19^ and was subsequently finalized through a standardized consultation process involving ten experts in the field of radiotherapy. The experts suggested including concurrent chemotherapy as a risk factor, as it may increase the risk of toxicity. They also recommended replacing the general term “malnutrition” with the more specific criterion of “BMI < 18.5 kg/m^2^”. Furthermore, considering the availability of routine clinical data, the LAR was excluded from the analysis as it is not part of the routine testing protocol for radiotherapy patients in our institution. Following these adjustments, the final set of potential risk factors for ARE determined in this study encompassed patient demographics, tumor-related factors, radiotherapy-related factors, other treatment-related factors, comorbidities, laboratory indices, and inflammatory markers, as shown in Fig. 2. Future studies are planned to further expand the range of variables through multi-center collaboration.

### Statistical analysis

In this study, a descriptive analysis of baseline patient characteristics was performed using SPSS 27.0. Categorical data are presented as frequencies and percentages (%).

The dependent variable was ARE occurrence. All variables showing statistical significance in the univariate analysis were included as independent variables for subsequent prediction model development. To leverage the specific strengths of different computational environments, models were constructed using the training set data with the following software: LR was implemented using SPSS 27.0, leveraging its robust generalized linear model module; the DT model was built using IBM SPSS Modeler 18.0, utilizing its intuitive and efficient visual data mining workflow; and the RF model was developed using R Studio, capitalizing on its powerful integrated learning packages (e.g., RF) to achieve high predictive accuracy and facilitate variable importance assessment. An identical set of predictor variables was used for all models to ensure a fair comparison.

After the models were constructed based on the training set, their predictive performances were evaluated and compared with those of the test set. The evaluation metrics included accuracy, precision, sensitivity, F1-score, and area under the receiver operating characteristic curve (AUC). Model calibration was performed using the Brier score and a calibration curve. The Brier score is the average squared distance between the predicted probability of the outcome and the true label; a lower Brier score indicates better model performance.

## Results

### Incidence of ARE among patients with cervical cancer undergoing radiotherapy

This study included 386 patients with cervical cancer undergoing radiotherapy, allocated to training (*n* = 270) and test (*n* = 116) sets, respectively. Among them, 204 (52.85%) developed ARE: 142 in the training set and 62 in the test set. The age range of the 386 patients was 28–78 years (54.90 ± 9.39). There were no statistically significant differences in baseline characteristics between the training and test sets (*P* > 0.05), and no statistically significant difference in the incidence of ARE between the two groups (*χ*^2^ = 0.024, *P* = 0.877), indicating that the indicators in both sets were relatively balanced (Appendix A).

### Univariate analysis of risk factors for ARE in patients with cervical cancer undergoing radiotherapy

The training set was divided into two groups based on the ARE development: an ARE-developing group (*n* = 142) and a non-ARE-developing group (*n* = 128). Univariate analysis was performed on general information, tumor-related factors, radiotherapy-related factors, other treatment-related factors, laboratory test indicators, and inflammation indicators for both groups. The analysis revealed statistically significant differences in age, clinical stage, parametrial tissue radiation dose, rectal V40, radiotherapy time, chemotherapy, hypertension, and PLR between the two groups (*P* < 0.05). The detailed results are presented in Table 2.

**Table 2:** Univariate analysis of risk factors for ARE in patients with cervical cancer undergoing radiotherapy [*N* = 270, *n* (%)].

Variables	Categories	The ARE-developing group (*n* = 142)	The non-ARE-developing group (*n* = 128)	*t/Z/χ*^2^	*P* value
Age (years)	< 60	72 (50.7)	92 (71.9)	12.653	< 0.001
	≥ 60	70 (49.3)	36 (28.1)		
BMI	< 18.5	18 (12.7)	12 (9.4)	0.202	0.653
(kg/m^2^)	18.5< 24.0	58 (40.8)	50 (39.1)		
	≥ 24.0	66 (46.5)	66 (51.5)		
Clinical stages	I	15 (10.6)	30 (23.4)	11.432	0.003
	II	38 (26.8)	41 (32.0)		
	III-IV	89 (62.7)	57 (44.5)		
Tumor diameter	≤ 4 cm	56 (39.4)	60 (46.9)	1.520	0.218
	> 4 cm	86 (60.6)	68 (53.1)		
Invasion depth	< 1/2	61 (43.0)	61 (47.7)	0.600	0.439
	≥ 1/2	81 (57.0)	67 (52.3)		
Radiotherapy time	07:00–11:00	67 (47.2)	33 (25.8)	13.353	0.001
	12:00–16:00	40 (28.2)	48 (37.5)		
	17:00–21:00	35 (24.6)	47 (36.7)		
Parametrial tissue	≤ 50 Gy	32 (22.5)	68 (53.1)	27.013	< 0.001
Radiation dose	> 50 Gy	110 (77.5)	60 (46.9)		
Rectal V40	< 40%	39 (27.5)	67 (52.3)	17.473	< 0.001
	≥ 40%	103 (72.5)	61 (47.7)		
No. of	≤ 10	49 (34.5)	37 (28.9)	4.118	0.128
Radiotherapy	11–20	52 (36.6)	39 (30.5)		
Sessions	21–28	41 (28.9)	52 (40.6)		
Radiotherapy	Supine	85 (59.9)	70 (54.7)	0.736	0.391
Position	Prone	57 (40.1)	58 (45.3)		
Chemotherapy	No	55 (38.7)	72 (56.3)	8.292	0.004
	Yes	87 (61.3)	56 (43.7)		
Hypertension	No	93 (65.5)	101 (78.9)	5.988	0.014
	Yes	49 (34.5)	27 (21.1)		
Anemia	No	114 (80.3)	100 (78.1)	0.190	0.663
	Yes	28 (19.7)	28 (21.9)		
Hyperglycemia	No	104 (73.2)	94 (73.4)	0.001	0.970
	Yes	38 (26.8)	34 (26.6)		
PLR	≤ 143	33 (23.2)	54 (42.2)	11.067	< 0.001
	> 143	109 (76.8)	74 (57.8)		
LMR	≤ 4	96 (67.6)	87 (68.0)	0.004	0.949
	> 4	46 (32.4)	41 (32.0)		

ARE, acute radiation enteritis; PLR, Platelet-to-Lymphocyte Ratio (calculated as the absolute platelet count divided by the absolute lymphocyte count); LMR, Lymphocyte-to-Monocyte Ratio (calculated as the absolute lymphocyte count divided by the absolute monocyte count).

### Prediction models for ARE risk in patients with cervical cancer undergoing radiotherapy

#### LR model

ARE data was coded (no ARE = 0, ARE occurrence = 1). Eight variables that exhibited statistical significance in univariate analysis were selected as independent variables for LR analysis. The assignments of the independent variables are presented in Table 3. The results indicated that age, clinical stage, parametrial tissue radiation dose, rectal V40, radiotherapy time, chemotherapy, and hypertension were significant factors influencing the occurrence of ARE in patients with cervical cancer undergoing radiotherapy (*P* < 0.05). These findings are summarized in Table 4.

**Table 3:** Multi-factor LR assignment.

Variables	Assignment case
Age	< 60 years = 0, ≥ 60 years = 1
Clinical stages	Ⅰ (0, 0), Ⅱ (1, 0), Ⅲ-Ⅳ (0, 1)
Parametrial tissue radiation dose	≤ 50 Gy = 0, > 50 Gy = 1
Rectal V40	< 40% = 0, ≥ 40% = 1
Radiotherapy time	07:00–11:00 (1, 0), 12:00–16:00 (0, 1), 17:00–21:00 (0, 0)
Chemotherapy	No = 0, yes = 1
Hypertension	No = 0, yes = 1
PLR	≤ 143 = 0, > 143 = 1

PLR, Platelet-to-Lymphocyte Ratio; LR, Logistic Regression.

**Table 4:** LR analysis results of ARE risk factors in patients with cervical cancer undergoing radiotherapy.

Variables	*β*	*SE*	Wald *χ*^2^	*P* value	*OR*	95% *CI*
Constant	−4.278	0.639	44.758	< 0.001	–	–
Age	0.984	0.312	9.934	0.002	2.674	1.451, 4.930
Clinical stages						
II	0.583	0.462	1.593	0.207	1.791	0.725, 4.429
III-IV	1.033	0.432	5.717	0.017	2.810	1.205, 6.554
Parametrial tissue radiation dose	1.749	0.335	27.224	< 0.001	5.749	2.980, 11.090
Rectal V40	1.005	0.307	10.689	0.001	2.731	1.495, 4.988
Radiotherapy time						
07:00–11:00	1.267	0.368	11.834	< 0.001	3.548	1.724, 7.302
12:00–16:00	0.130	0.371	0.124	0.725	1.139	0.551, 2.357
Chemotherapy	0.867	0.304	8.122	0.004	2.380	1.311, 4.321
Hypertension	0.819	0.347	5.575	0.018	2.269	1.149, 4.480
PLR	0.570	0.321	3.155	0.076	1.768	0.943, 3.314

ARE, acute radiation enteritis; PLR, Platelet-to-Lymphocyte Ratio; CI, Confidence Interval; SE, Standard Error; OR, odds ratio; LR, Logistic Regression.

#### DT model

Eight predictive variables identified through univariate analysis were incorporated into the DT model. The resulting tree structure consisted of five layers and 22 nodes, including 12 terminal nodes. The model identified six key explanatory variables using recursive partitioning. Specifically, the parametrial tissue radiation dose, which served as the root node variable, was the primary predictor of ARE in patients with cervical cancer undergoing radiotherapy. Age and hypertension were positioned at the second level and functioned as secondary risk stratification indicators. Rectal V40 was located at the third level, radiotherapy time was at the fourth level, and PLR was at the fifth level.

#### RF model

**Fig. 3:**
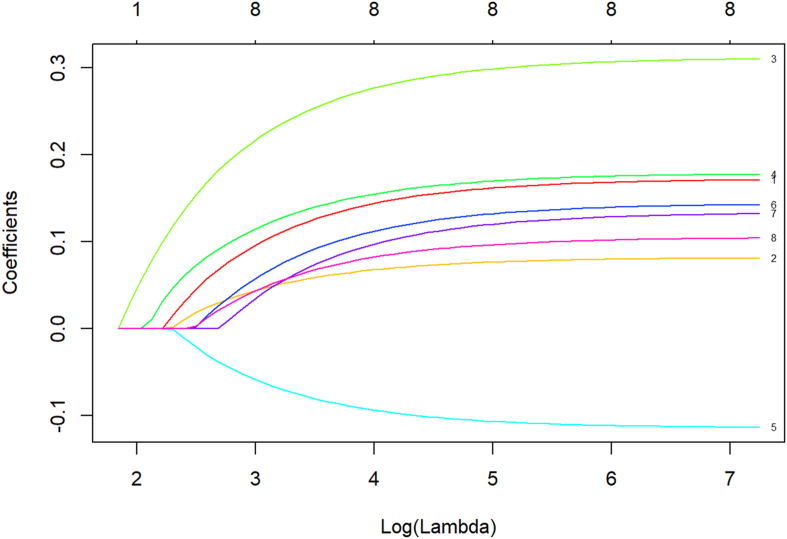
Feature selection based on LASSO analysis. LASSO, Least Absolute Shrinkage and Selection Operator.

**Fig. 4:**
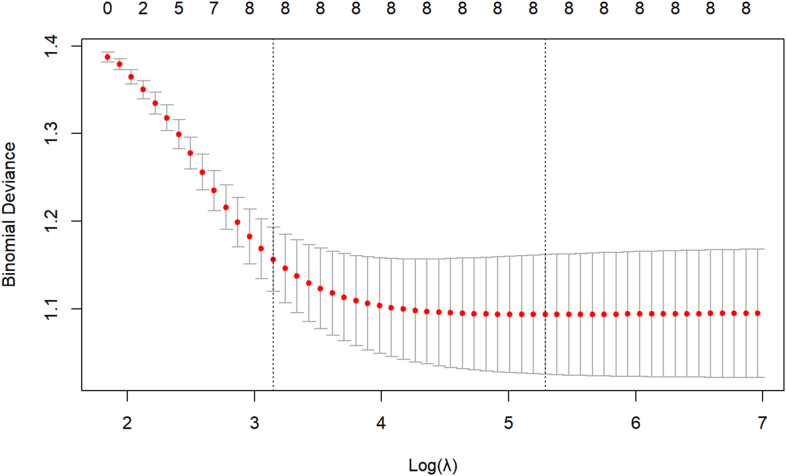
LASSO regression with cross-validation. LASSO, Least Absolute Shrinkage and Selection Operator.

**Fig. 5:**
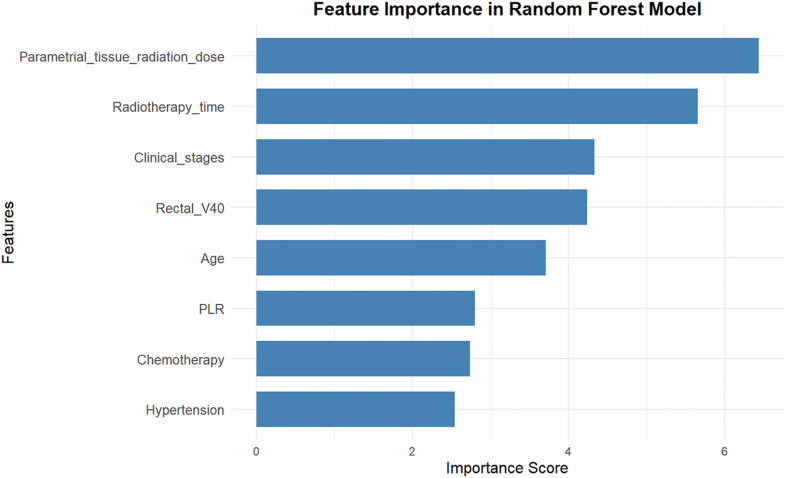
Ranking of factors influencing ARE. ARE, acute radiation enteritis; PLR, Platelet-to-Lymphocyte Ratio.

In R Studio, the *glmnet* function was employed to conduct a Least Absolute Shrinkage and Selection Operator (LASSO) regression analysis of the eight variables that demonstrated statistical significance in the single-factor analysis, as shown in Fig. 3, Fig. 4. The vertical dashed line on the left indicates *lambda.min*, whereas the straight line on the right represents *lambda.1se* value. When the *lambda* (λ) value is 0.005053, the model achieves the minimum error, corresponding to the eight influencing factors. Parametrial tissue radiation dose, radiotherapy time, clinical stage, rectal V40, age, PLR, chemotherapy, and hypertension were identified as risk factors for ARE in patients with cervical cancer undergoing radiotherapy. The ranking of variable importance is shown in Fig. 5.

### Comparison of predictive performance of three risk prediction models

**Fig. 6:**
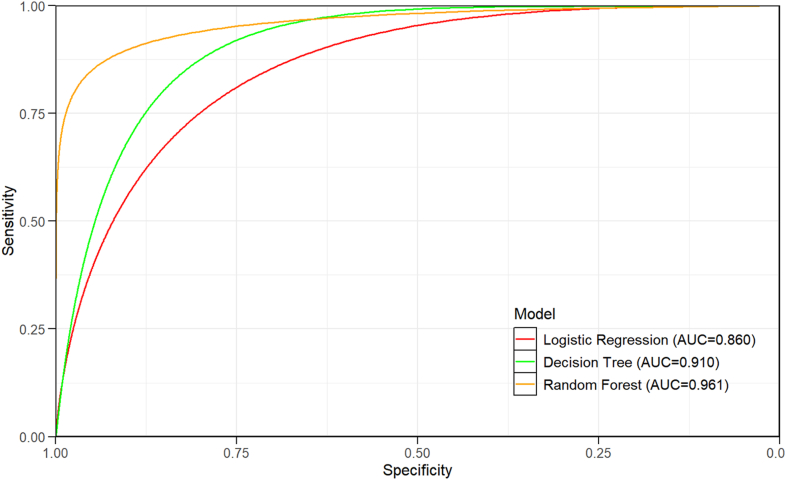
ROC curves for performance comparison of three predictive models. ROC, receiver operating characteristic; AUC, area under the curve.

**Fig. 7:**
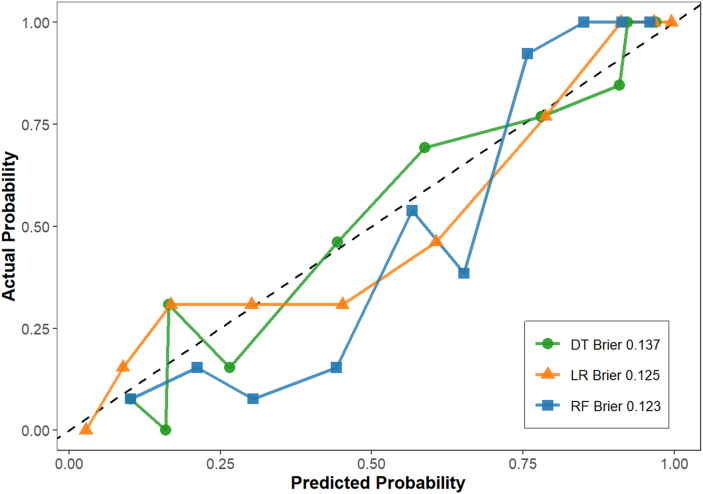
Calibration curve plots of three prediction models. LR, Logistic Regression; DT, Decision Tree; RF, Random Forest.

The performance evaluation results of the three models for the test set are detailed in Table 5. As shown in the table, the RF model performed optimally across all evaluation metrics, with higher accuracy, precision, sensitivity, F1 score, and AUC values than the DT and LR models. Although all three models exhibited good predictive performances (AUC > 0.85), the RF model demonstrated the strongest comprehensive predictive performance. The ROC curves for each model are shown in Fig. 6. The Brier score of the RF model in predicting delirium was 0.123 in the validation cohort, indicating that the model was reliable. The calibration curves for the model are shown in Fig. 7.

**Table 5:** Comparison of predictive performance of the three models on the test set.

Model	Accuracy	Precision	Sensitivity	F1-Score	AUC	95% CI
Logistic regression	0.722	0.734	0.739	0.736	0.860	0.818, 0.882
Decision tree	0.862	0.859	0.887	0.873	0.910	0.846, 0.900
Random forest	0.897	0.877	0.934	0.905	0.961	0.867, 0.918

AUC, area under the receiver operating characteristic curve; CI, Confidence Interval.

## Discussion

### Main findings

Radiotherapy is a common clinical treatment for cervical cancer, effectively prolonging patient survival. Symptoms like hematochezia, abdominal pain, and diarrhea during radiotherapy significantly affect patient prognosis.^5^ In this study, the incidence of ARE in patients with cervical cancer undergoing radiotherapy was 52.85%, which is lower than the 65.20% reported in the literature,^7^ but still relatively high. This not only limits radiotherapy dose escalation and delays treatment but also risks severe complications such as intestinal fibrosis and obstruction if not managed promptly.^5^ Thus, enhancing nursing management and closely monitoring treatment side effects are urgently required.

Before radiotherapy, patients should be educated on disease knowledge, treatment procedures, and dietary advice. Specialized assessments, including intestinal symptom evaluation, and guidance on radiotherapy positioning and cooperation are essential. Individualized hydration and precise bladder filling training can improve bladder stability and clinical efficacy, reducing ARE risk.^33^^,^^34^ Timely interventions are crucial when symptoms arise, such as traditional Chinese medicine retention enemas to alleviate ARE symptoms,^35^ and nutritional support to improve nutritional and immune status, reduce digestive juice secretion, and relieve abdominal pain and diarrhea.^36^ Comprehensive treatment evaluation is also vital, involving timely identification of high-risk patients and early detection of ARE signs to prevent and reduce its incidence, thereby alleviating patient suffering.

This study identified several key risk factors for ARE in patients with cervical cancer undergoing radiotherapy, including parametrial tissue radiation dose, age, hypertension, rectal V40, and radiotherapy time. These factors were consistently identified across the three predictive models (LR, DT, and RF), highlighting their significance in ARE prevention.

The parametrial tissue radiation dose (> 50 Gy) and rectal V40 (≥ 40%) emerged as critical risk factors for ARE, consistent with the findings of Xu et al.^14^ and Chen et al.^19^ In the DT model, the parametrial tissue radiation dose served as the root node, whereas, in the RF model, it was the most important factor. Rectal V40 was positioned in the third layer of the DT model and ranked fourth in the RF model. This is likely because the small intestine and the rectum are the main organs exposed to radiation during radiotherapy for CC. When the cumulative dose of external pelvic radiation exceeds 50 Gy, the risk of acute intestinal reactions significantly increases.^37^ A rectal V40 of ≥ 40% indicates extensive exposure of the rectum to high-dose radiation, leading to severe damage and increased ARE risk.^14^^,^^38^ Nurses should collaborate with radiation therapists to ensure precise radiotherapy targeting and implement preventive measures, such as administering amifostine retention enemas to protect the intestinal mucosa and promote repair,^39^ especially for patients receiving high-dose radiotherapy.

Age was a significant predictor of the ARE, ranking second and fifth in the DT and RF models, respectively. In this study, the incidence of ARE in patients with cervical cancer aged 60 years or older was 1.548 times higher than that in patients aged < 60 years. This is consistent with the results of previous studies.^16^ This may be attributed to age-related declines in bodily and gastrointestinal functions as well as pelvic floor muscle relaxation in elderly women, leading to poor vaginal extensibility. This results in limited protective packing during brachytherapy, which significantly increases the risk.^10^^,^^40^ Although age is an uncontrollable factor, nurses can enhance symptom monitoring in elderly patients and guide them in pelvic floor muscle exercises to strengthen these muscles, thereby mitigating the risk of ARE.

Hypertension was another notable risk factor for ARE, ranking second and eighth in the DT and RF models, respectively. The Chinese Expert Consensus on Diagnosis and Management of Radiation Proctitis (2018 Edition) also suggested that hypertension is associated with a higher risk of ARE,^41^ which aligns with the findings of this study. The underlying mechanism may involve long-term hypertension, which causes intestinal microvascular and capillary damage,^7^ leading to chronic local blood supply deficiency and delayed repair of the radiation-damaged intestinal mucosa. Additionally, hypertension is associated with chronic systemic inflammation,^42^ which can disrupt the balance of the intestinal flora, weaken the body's anti-inflammatory response, and induce ARE. Clinical nursing staff should closely monitor patients with hypertension during radiotherapy, provide tailored dietary and exercise guidance, control blood pressure, and promptly address symptoms, such as diarrhea, to prevent ARE.

Radiotherapy time was positioned at the fourth level in the DT model and ranked second in the RF model. This study found that patients who received radiotherapy in the morning had a higher risk of ARE, which is consistent with a previous multi-center randomized controlled trial.^18^ This may be due to the circadian rhythm of intestinal crypt cell apoptosis, which peaks during the morning.^43^, ^44^, ^45^, ^46^ Therefore, scheduling pelvic radiotherapy for high-risk patients during the rest of the day whenever possible is advisable. If morning radiotherapy is necessary, increased monitoring and prompt treatment of early ARE symptoms are essential to reduce toxicity and risk.

Clinical staging and concurrent chemotherapy were identified as predictive factors in both the LR and RF models, whereas PLR was identified using both the DT and RF models. Late-stage clinical stages (III–IV) increase the radiation exposure area and dose to the normal intestine, whereas advanced disease stages are associated with poor physical condition, low immunity, and weak intestinal repair ability, exacerbating intestinal damage.^13^^,^^47^ Concurrent chemoradiotherapy doubles the incidence of grade 3–4 gastrointestinal toxicity compared to radiotherapy alone,^48^ likely due to the synergistic effects and cumulative toxicity of the two treatments. PLR, an indicator of systemic inflammation and immune response, can be used to assess the severity of AREs.^19^^,^^49^^,^^50^ Nurses should closely monitor gastrointestinal reactions in patients with stage III–IV disease and those undergoing concurrent chemotherapy, adjust dietary and chemotherapy regimens as needed, and provide comprehensive measures, such as anti-inflammatory and immune regulation, for patients with high PLR levels to reduce the risk of ARE and promote recovery.

The RF model outperformed the other models examined in this study, demonstrating superior predictive accuracy on the test set compared with the LR and DT models, which is consistent with prior clinical outcome prediction research.^51^, ^52^, ^53^ While LR allows for the visual representation of risk factors via Odds Ratios (OR), it requires a large sample size and balanced data distribution, and is sensitive to missing data, potentially compromising model stability.^54^ DT offers good visualization and can handle missing data; however, its complex tree structure is prone to overfitting, which limits its generalizability.^55^ In contrast, the RF model, as an ensemble learning algorithm, reduces individual biases by integrating multiple decision trees and automatically accounting for variable interactions and nonlinear effects. Its Bootstrap sampling mechanism ensures efficient data utilization and adaptability to diverse data types, enhancing prediction accuracy and yielding more precise, stable, and robust outcomes.^56^^,^^57^ Therefore, the RF model is considered the most effective for predicting the risk of ARE in patients with cervical cancer undergoing radiotherapy and is the optimal choice for this research context.

### Implications for nursing practice and research

The predictive model developed in this study has significant implications for nursing practice and research. It provides oncology nurses with a practical tool to objectively stratify patients’ risk of developing ARE prior to radiotherapy. This enables targeted nursing assessment, early intervention, and personalized patient education. For instance, high-risk patients can be proactively counselled regarding symptom management and dietary modifications, and nursing care plans can be intensified for closer monitoring. For future research, this study established a validated foundation for developing clinical decision support systems embedded within electronic health records. Further investigations should focus on the implementation and impact of applying this model in real-world clinical workflows to evaluate its effectiveness in improving patient outcomes and alleviating the ARE symptom burden.

### Limitations

This study had certain limitations. First, this was a single-center prospective cohort study with all data from a single tertiary hospital. The representativeness of the sample may have been affected by geographical factors, population characteristics, and diagnostic and treatment processes. Despite internal validation, the lack of external validation across different healthcare environments limits widespread clinical application. Second, patients with significant medical histories or impaired vital organ function were excluded from the study, which may have led to selection bias and potentially reduced the predictive performance of the model in populations with poor physical condition. In addition, the exclusion of potential influencing factors may also lead to residual confounding bias. In terms of model interpretability, random forest is a more complex machine learning model. Although it performs better in terms of predictive performance, the integration of multiple decision trees renders the internal decision-making process of the model difficult to interpret intuitively, thereby limiting its interpretability. Future research should expand the sample source through multi-center collaboration, conduct external validation, and explore more interpretable machine learning methods to improve the model's generalization ability and clinical operability. Simultaneously, other potential factors that may influence ARE in patients with cervical cancer undergoing radiotherapy should be investigated to continuously refine the model, thereby offering more reliable and targeted evidence for preventing ARE in this population in clinical practice.

## Conclusions

Machine learning has opened a new path for research on the risk and factors influencing ARE in patients with cervical cancer undergoing radiotherapy. This study utilized an RF model constructed through machine learning, which outperformed the DT and LR models in terms of predictive performance. This study revealed that the risk of ARE in patients with cervical cancer undergoing radiotherapy is influenced by various factors, including parametrial tissue radiation dose, rectal V40, age, hypertension, radiotherapy time, clinical stage, chemotherapy, and PLR. Based on these findings, clinical healthcare professionals can quickly and accurately identify individuals at a high risk of ARE, thereby enabling the development of timely, predictable, and personalized intervention plans for high-risk patients. Future research should focus on conducting multi-center, large-sample, prospective studies to validate and optimize the predictive models. The ultimate goal is to integrate them into clinical decision support systems to assist clinical decision-making through real-time risk alerts, thereby achieving precision medicine and improving patient outcomes.

## CRediT authorship contribution statement

**Zhao Wang:** Conceptualization, Methodology, Software, Data curation, Writing–Original Draft. **Huiying Liu:** Data curation, Writing–Original Draft. **Xiaocen Chen:** Writing–Review & Editing. **Fang Zhang:** Investigation. **Yixuan Liu:** Investigation. **Jiayun Sun:** Investigation. **Lili Liu:** Software, Validation. **Xiaotong Yang:** Software, Validation. All authors have read and approved the final manuscript.

## Ethics statement

The study was approved by the Institutional Review Board of the Tianjin Medical University Cancer Institute & Hospital (Approval No. bc20240022) and was conducted in accordance with the 1964 Helsinki Declaration and its later amendments or comparable ethical standards. All participants provided written informed consent.

## Data availability statement

The data that support the findings of this study are available on request from the corresponding author, XC. The data are not publicly available due to privacy or ethical restrictions.

## Declaration of generative AI and AI-assisted technologies in the writing process

No AI tools/services were used during the preparation of this work.

## Funding

This study was supported by Tianjin Key Medical Discipline Construction Project (Grant No. TJYXZDXK-3-004B); Tianjin Medical University Cancer Institute & Hospital Nursing Special Fund Project (Grant No. H2304). The funders had no role in considering the study design or in the collection, analysis, interpretation of data, writing of the report, or decision to submit the article for publication.

## Declaration of competing interest

The authors declare no conflict of interest.
