# Supplementary material for: Comparative predictive performance of three machine learning algorithms for acute radiation enteritis risk among patients with cervical cancer undergoing radiotherapy: A prospective cohort study
Source: Asia Pac J Oncol Nurs. 2026 Jan 17;13:100853. doi: 10.1016/j.apjon.2026.100853 (PMC12874331; doi:10.1016/j.apjon.2026.100853)
Supplement: Multimedia component 1 [file mmc1.docx]

**Appendix A.**

**Comparison of baseline characteristics between patients in the training set and the test set**

| Variables | Categories | Training set  (*n* = 270) | Test set  (*n* = 116) | *t/Z/χ*^2^ | *P* |
| --- | --- | --- | --- | --- | --- |
| RE | No | 128 (47.4) | 54 (46.6) | 0.024 | 0.877 |
|  | Yes | 142 (52.6) | 62 (53.4) |  |  |
| Age | < 60 years | 164 (60.7) | 71 (61.2) | 0.007 | 0.931 |
|  | ≥ 60 years | 106 (39.3) | 45 (38.8) |  |  |
| BMI | <18.5 | 30 (11.1) | 18 (15.5) | 1.470 | 0.480 |
| (kg/m^2^) | 18.5-<24 | 108 (40.0) | 45 (38.8) |  |  |
|  | ≥ 24 | 132 (48.9) | 53 (45.7) |  |  |
| Clinical stages | Ⅰ | 45 (16.7) | 17 (14.7) | 0.291 | 0.865 |
|  | Ⅱ | 79 (29.2) | 36 (31.0) |  |  |
|  | Ⅲ~Ⅳ | 146 (54.1) | 63 (54.3) |  |  |
| Tumor diameter | ≤ 4 cm | 116 (43.0) | 47 (40.5) | 0.199 | 0656 |
|  | > 4 cm | 154 (57.0) | 69 (59.5) |  |  |
| Invasion depth | < 1/2 | 122 (45.2) | 53 (45.7) | 0.008 | 0.927 |
|  | ≥ 1/2 | 148 (54.8) | 63 (54.3) |  |  |
| Radiotherapy time | 07:00-11:00 | 100 (37.0) | 54 (46.6) | 3.256 | 0.196 |
|  | 12:00-16:00 | 88 (32.6) | 30 (25.9) |  |  |
|  | 17:00-21:00 | 82 (30.4) | 32 (27.6) |  |  |
| Parametrial tissue | ≤ 50 Gy | 100 (37.0) | 42 (36.2) | 0.024 | 0.877 |
| radiation dose | > 50 Gy | 170 (63.0) | 74 (63.8) |  |  |
| Rectal V40 | < 40% | 106 (39.3) | 51 (44.0) | 0.745 | 0.388 |
|  | ≥ 40% | 164 (60.7) | 65 (56.0) |  |  |
| Number of | ≤ 10 | 86 (31.9) | 35 (30.2) | 0.106 | 0.948 |
| radiotherapy | 11~20 | 91 (33.7) | 40 (34.5) |  |  |
| sessions | 21~28 | 93 (34.4) | 41 (35.3) |  |  |
| Radiotherapy | Supine | 155 (57.4) | 67 (57.8) | 0.004 | 0.949 |
| position | Prone | 115 (42.6) | 49 (42.2) |  |  |
| Chemotherapy | No | 127 (47.0) | 50 (43.1) | 0.506 | 0.477 |
|  | Yes | 143 (53.0) | 66 (56.9) |  |  |
| Hypertension | No | 194 (71.9) | 80 (69.0) | 0.328 | 0.567 |
|  | Yes | 76 (28.1) | 36 (31.0) |  |  |
| Anemia | No | 214 (79.3) | 94 (81.0) | 0.159 | 0.690 |
|  | Yes | 56 (20.7) | 22 (19.0) |  |  |
| Hyperglycemia | No | 198 (73.3) | 87 (75.0) | 0.117 | 0.733 |
|  | Yes | 72 (26.7) | 29 (25.0) |  |  |
| PLR | ≤ 143 | 87 (32.2) | 36 (31.0) | 0.053 | 0.818 |
|  | > 143 | 183 (67.8) | 80 (69.0) |  |  |
| LMR | ≤ 4 | 183 (67.8) | 77 (66.4) | 0.072 | 0.788 |
|  | > 4 | 87 (32.2) | 39 (33.6) |  |  |
